# Bidirectional Causal Associations Between Endogenous/Exogenous Antioxidant Levels and Risks of Type 1 and Type 2 Diabetes Mellitus and Three Complications

**DOI:** 10.1002/fsn3.70671

**Published:** 2025-07-24

**Authors:** Liang Shen, Lei Meng, Hong‐Fang Ji

**Affiliations:** ^1^ Institute of Food and Drug Research for One Health, School of Food Engineering Ludong University Yantai People's Republic of China; ^2^ School of Life Sciences and Medicine Shandong University of Technology Zibo People's Republic of China

**Keywords:** albumin, bilirubin, diabetes mellitus, Mendelian randomization

## Abstract

Owing to the significant pathogenic role played by oxidative stress in diabetes mellitus, the associations between antioxidants and the incidence of diabetes mellitus have garnered much interest, while the findings are conflicting. The present study aimed to investigate the bidirectional causal connections underlying the relationship between circulating levels of eight endogenous and five exogenous antioxidants and the risks of type 1 diabetes mellitus (T1DM) and type 2 diabetes mellitus (T2DM), as well as three complications: diabetic ketoacidosis, diabetic nephropathy, and diabetic retinopathy, by means of Mendelian randomization analyses. The analyses indicate that albumin and bilirubin may causally contribute to protection against the development of T1DM and T2DM, respectively. The exogenous β‐carotene is likely to act as a protective factor against the development of T2DM. Bilirubin may have a causally protective role in preventing the development of diabetic ketoacidosis. For the reverse analysis of diseases predicting antioxidant levels, T1DM, T2DM, and their complications were likely to be associated with varied levels of several antioxidants, but the effects are weak overall. Our analyses may provide useful clues that inform the use of antioxidants for preventing or predicting both diabetes mellitus and its complications.

## Graphical abstract

The bidirectional causal associations between levels of a series of *endogenous* and exogenous antioxidants and risks of type 1 diabetes mellitus (T1DM) and type 2 diabetes mellitus (T2DM), as well as three complications, diabetic ketoacidosis, diabetic nephropathy, and diabetic retinopathy, were investigated by Mendelian randomization analyses.

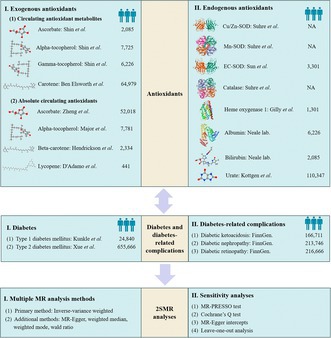

## 1 Introduction

Due to the increasing prevalence worldwide, diabetes mellitus now represents one of the main hazards to public health problems (Gregory et al. 2022; NCD Risk Factor Collaboration 2016; Cho et al. 2018). Diabetes mellitus (DM), which is characterized by increased blood sugar levels, falls primarily into two types: type 1 diabetes mellitus (T1DM) and type 2 diabetes mellitus (T2DM) (ElSayed et al. 2023). The former arises from the lack of insulin generated by pancreatic beta cells, and the latter is caused by impaired insulin secretion or insulin resistance. In addition, mismanaged DM can cause a series of dangerous complications, like diabetic ketoacidosis (DK), diabetic nephropathy (DN), and diabetic retinopathy (DR) (Ali et al. 2022).

Accumulating evidence from experimental research and clinical practice in the past decades supports oxidative stress triggered by abnormally elevated reactive oxygen species (ROS) concentrations, in the pathogenesis of both T1DM and T2DM (Rains and Jain 2011; Brownlee 2005; Henriksen et al. 2011). The antioxidant defense system is of critical importance to balance ROS levels and prevent oxidative stress damage. The endogenous and exogenous antioxidants constitute the antioxidant defense system. The known macromolecular antioxidants mainly include enzymatic molecules (like superoxide dismutase (SOD), heme oxygenase 1 (HO‐1), catalase), and non‐enzymatic ones (like uric acid, bilirubin, and albumin). The known dietary micromolecular antioxidants mainly include ascorbate, vitamin E, β‐carotene, and etc.

Despite many studies investigating the effects of antioxidants against DM and its complications, the conclusions are not always consistent. Many studies have reported that higher plasma carotenoids and vitamin C levels were linked to a lower risk of diabetes onset (Harding et al. 2008; Coyne et al. 2005). In comparison, intervention studies offering supplements of antioxidant vitamins have demonstrated conflicting findings to lower the incidence of diabetes or its complications (Abdali et al. 2015; Liu et al. 1999; Kataja‐Tuomola et al. 2011). With the aim of providing clues to expand therapeutic options, it is of importance to estimate the relationships between antioxidants and DM as well as its complications.

**FIGURE 1:**
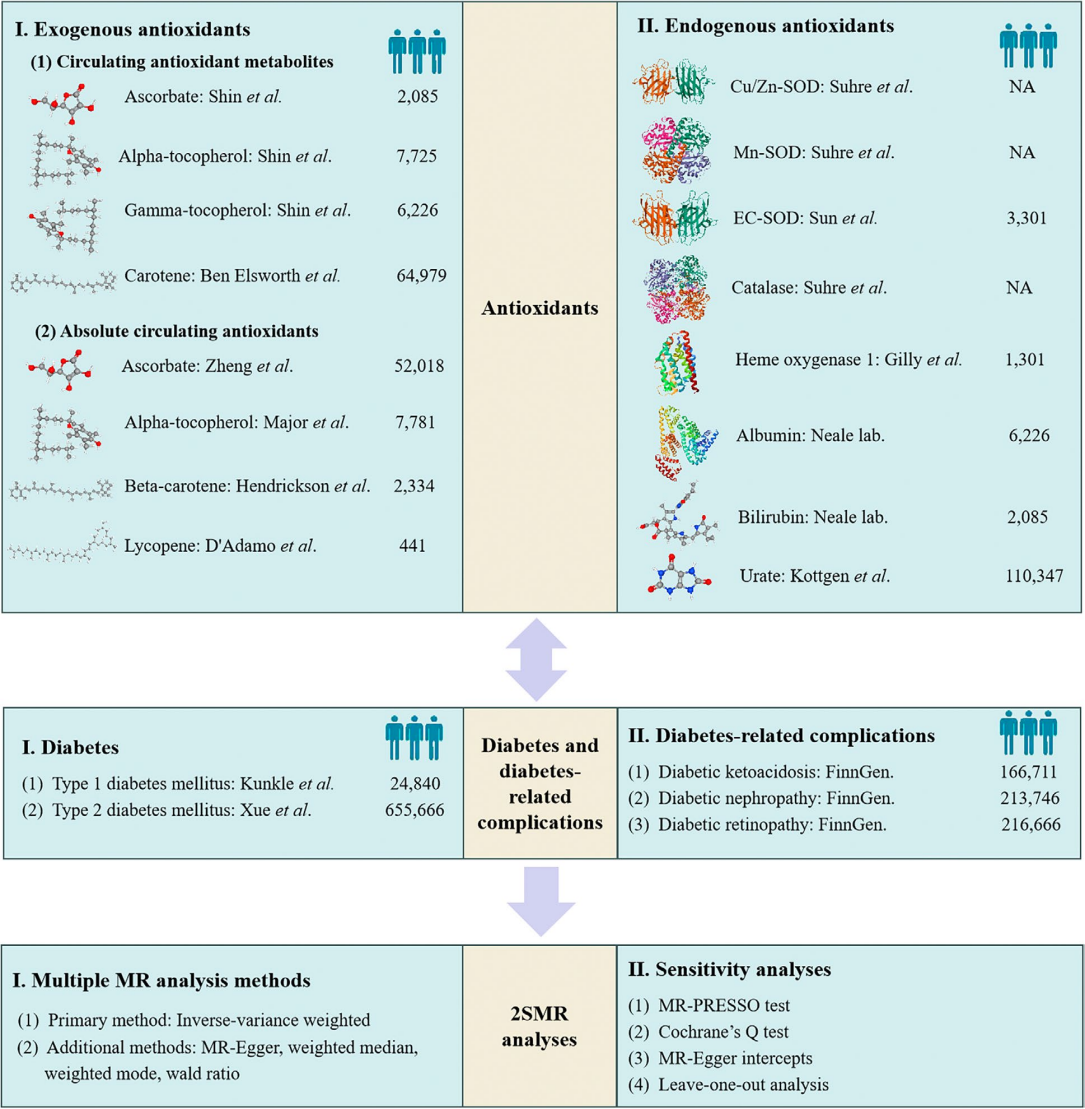
Schematic representation of the MR analysis of the bidirectional causal association between endogenous and exogenous antioxidant levels and risks of T1DM, T2DM, and its complications. The first section of the diagram illustrated the GWAS related to antioxidants included in this study. A total of eight endogenous and five exogenous antioxidants (comprising absolute circulating exogenous antioxidants and circulating antioxidant metabolites levels) were involved in the 2SMR analyses. The second section encompassed genetic data associated with T1DM, T2DM, and three complications (DK, DN, DR) derived from GWAS conducted in individuals of European ancestry. Both the first and second sections provided an overview of the essential characteristics of exposures and outcomes, including information about authors and sample sizes. In the current bidirectional MR analysis, the forward MR analysis used antioxidant levels as the exposures and diabetes and its complications as the outcomes, while the reverse MR analysis employed diabetes and its complications as the exposures and antioxidant levels as the outcomes. The third section described the primary methods utilized in the MR analysis and sensitivity analyses, including IVW, weighted median, weighted mode, MR‐Egger regression, MR‐PRESSO test, Cochrane's Q test, MR‐Egger intercepts, and leave‐one‐out analysis.

As Mendelian randomization (MR) can overcome limitations associated with observational studies such as confounding effects or reverse causation, in recent years it has been widely employed to assess the causal associations between various types of exposures and diseases (Papadimitriou et al. 2020; Liu et al. 2019; Hu et al. 2022; Meng, Wang, Ji, and Shen 2022; Meng, Wang, Ming, et al. 2022; Ren et al. 2023), including DM (Manousaki et al. 2021; Chen et al. 2023; Mordi et al. 2021; Zheng et al. 2021; Martin et al. 2022). In the current study, based on two‐sample MR (2SMR) analyses, we aim to fully analyze the causal associations between circulating levels of endogenous and exogenous antioxidants and risks of T1DM and T2DM, as well as three complications, DK, DN, and DR. The endogenous antioxidants considered here include Cu/Zn‐SOD, Mn‐SOD, extracellular (EC)‐SOD, catalase, HO‐1, bilirubin, albumin, and uric acid. As to exogenous ones, both absolute antioxidant levels and antioxidant metabolites of ascorbate, α‐tocopherol, γ‐tocopherol, β‐carotene, and lycopene are included. The second aim of our study was to investigate the association between T1DM, T2DM, and its complications in predicting antioxidant levels for the first time (Figure 1).

## 2 Methods

All available instrumental variables involved in the study were obtained from the published literature and public IEU‐GWAS database. The TwoSample MR package 0.5.6 provided by the MR‐base platform was used to retrieve, filter, and extract the summary data from GWAS, and conduct 2SMR analysis automatically (Hemani et al. 2018). For the genetic data of antioxidants of eight endogenous ones including superoxide dismutase (Cu/Zn‐SOD, Mn‐SOD, and EC‐SOD), catalase, HO‐1, bilirubin, urate, albumin, and exogenous antioxidants including absolute circulating exogenous antioxidants as well as circulating antioxidant metabolites of ascorbate, lycopene, carotene, α‐tocopherol, and γ‐tocopherol, were involved in the 2SMR analyses (Zheng et al. 2021; Shin et al. 2014; Suhre et al. 2017; Köttgen et al. 2013; Gilly et al. 2020; Sun et al. 2018; Hendrickson et al. 2012; D'Adamo et al. 2016; Major et al. 2011). As for diseases, the genetic data of two main types of diabetes (T1DM, T2DM) and its complications (DK, DN, DR) was derived from the GWAS among European‐ancestry populations (Forgetta et al. 2020; Xue et al. 2018).

To identify instrumental variables exhibiting a reliable association with the exposure, SNPs with strong correlation (*p* < 5 × 10^−8^) and without any link disequilibrium (clump_r2 = 0.001, clump_kb = 10,000) were chosen to summarize the data. The conventional inverse variance weighting (IVW) method was usually used as the primary method for 2SMR analysis; weighted median, weighted mode, and MR‐Egger regression were employed as additional methods to evaluate the accuracy of the analyses. Final causal effects were considered to be statistically significant at *p*‐value < 0.05.

The stability and accuracy of 2SMR results were verified via sensitivity analyses. First of all, MR‐PRESSO was employed to detect outliers that affected the stability of the results (Verbanck et al. 2018). If there were outliers identified by the MR‐PRESSO test, we would exclude them and conduct a causal analysis again with the remaining ones. In addition, Cochran's Q statistics and MR‐Egger intercept test were performed for heterogeneity and pleiotropy analyses. Further leave‐one‐outcome analyses were also utilized to examine the impact of individual SNPs on the overall causal effect. The schematic representation of MR analysis in this paper is shown in Figure 1.

## 3 Results

### 3.1 Outcomes of the Forward 2SMR Analyses

#### 3.1.1 Effect of Endogenous and Exogenous Antioxidant Levels on Risk of T1DM

**FIGURE 2:**
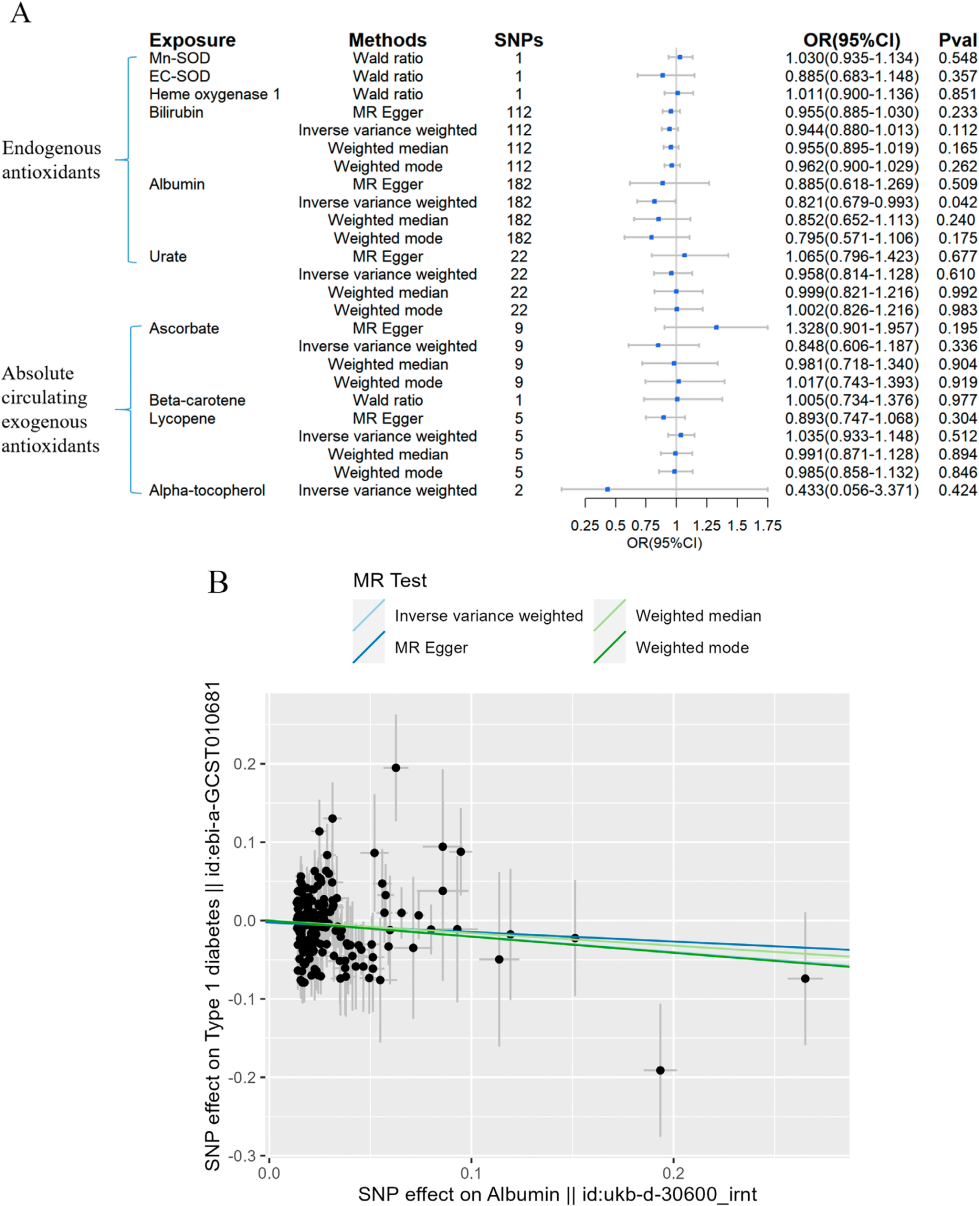
Causal estimates of forward 2SMR analyses between antioxidant levels and risks of T1DM. (A) The forest plot demonstrated the results of the forward 2SMR analysis between T1DM and absolute circulating antioxidants, and endogenous antioxidants, respectively. The OR for the results is depicted by the blue box of the forest plot, with the vertical lines representing the null effect (OR = 1) and the horizontal line representing the confidence interval. (B) The scatter plot displayed a causal association between increased albumin levels and a reduced risk of T1DM, and the slopes of colored lines correspond to the causal effects estimated through different 2SMR analysis methods.

**FIGURE 3:**
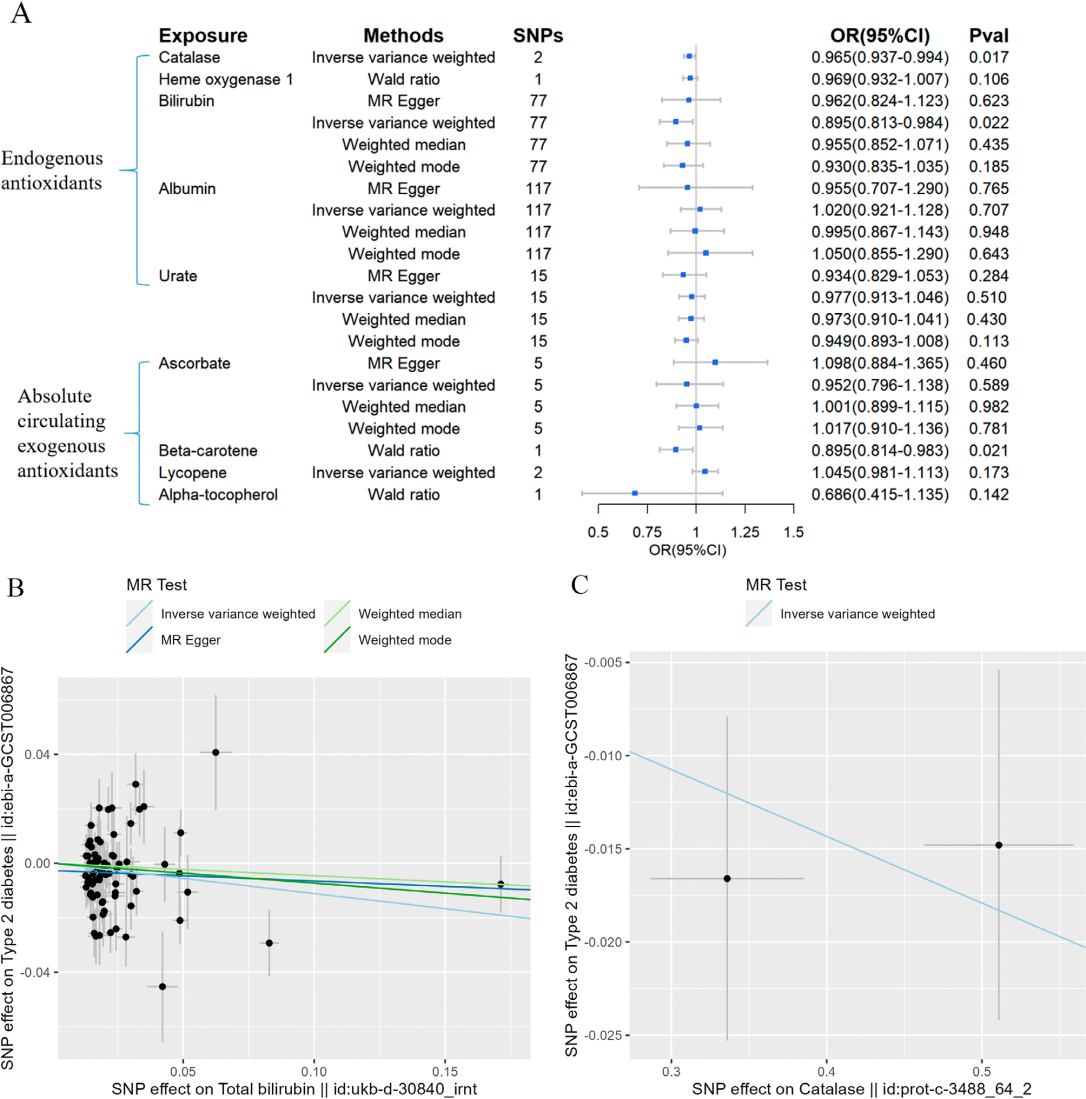
Causal estimates of forward 2SMR analyses between antioxidant levels and risks of T2DM. (A) The forest plot demonstrated the results of the forward 2SMR analysis between T2DM and absolute circulating antioxidants, and endogenous antioxidants, respectively. The OR for the results is depicted by the blue box of the forest plot, with the vertical lines representing the null effect (OR = 1) and the horizontal line representing the confidence interval. (B) The scatter plot displayed a causal association between increased bilirubin levels and a decreased risk of T2DM, and the slopes of colored lines correspond to the causal effects estimated through different 2SMR analysis methods. (C) The scatter plot displayed a causal relationship between increased catalase concentrations and a decreased risk of T2DM, and the slopes of colored lines correspond to the causal effects estimated through different 2SMR analysis methods.

**FIGURE 4:**
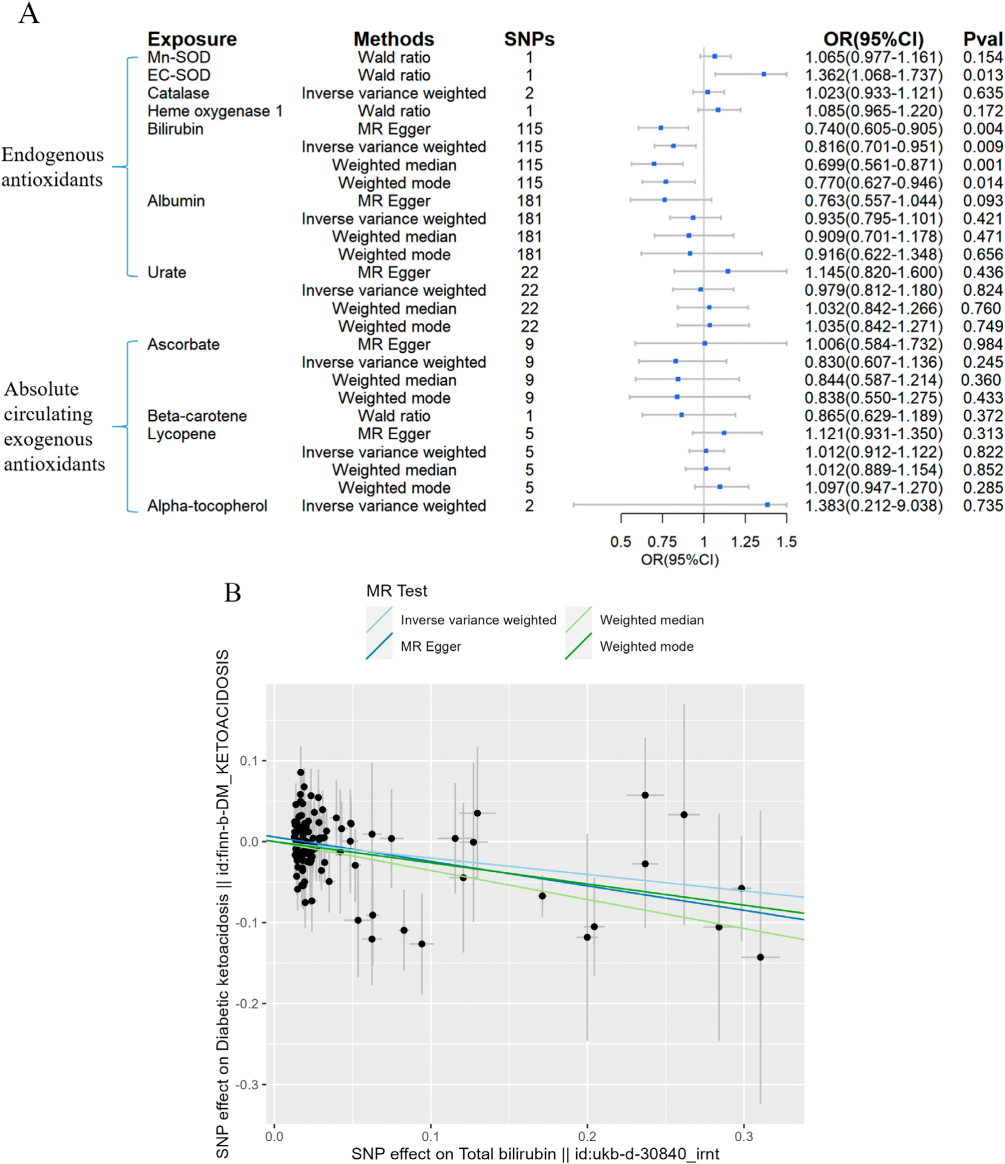
Causal estimates of forward 2SMR analyses between antioxidant levels and risks of DK. (A) The forest plot demonstrated the 2SMR results between DK and absolute circulating antioxidants, and endogenous antioxidants, respectively. The OR for the results is depicted by the blue box of the forest plot, with the vertical lines representing the null effect (OR = 1) and the horizontal line representing the confidence interval. (B) The scatter plot illustrated a causal relationship between elevated bilirubin levels and a reduced risk of DK, and the slopes of colored lines correspond to the causal effects estimated through different 2SMR analysis methods.

**FIGURE 5:**
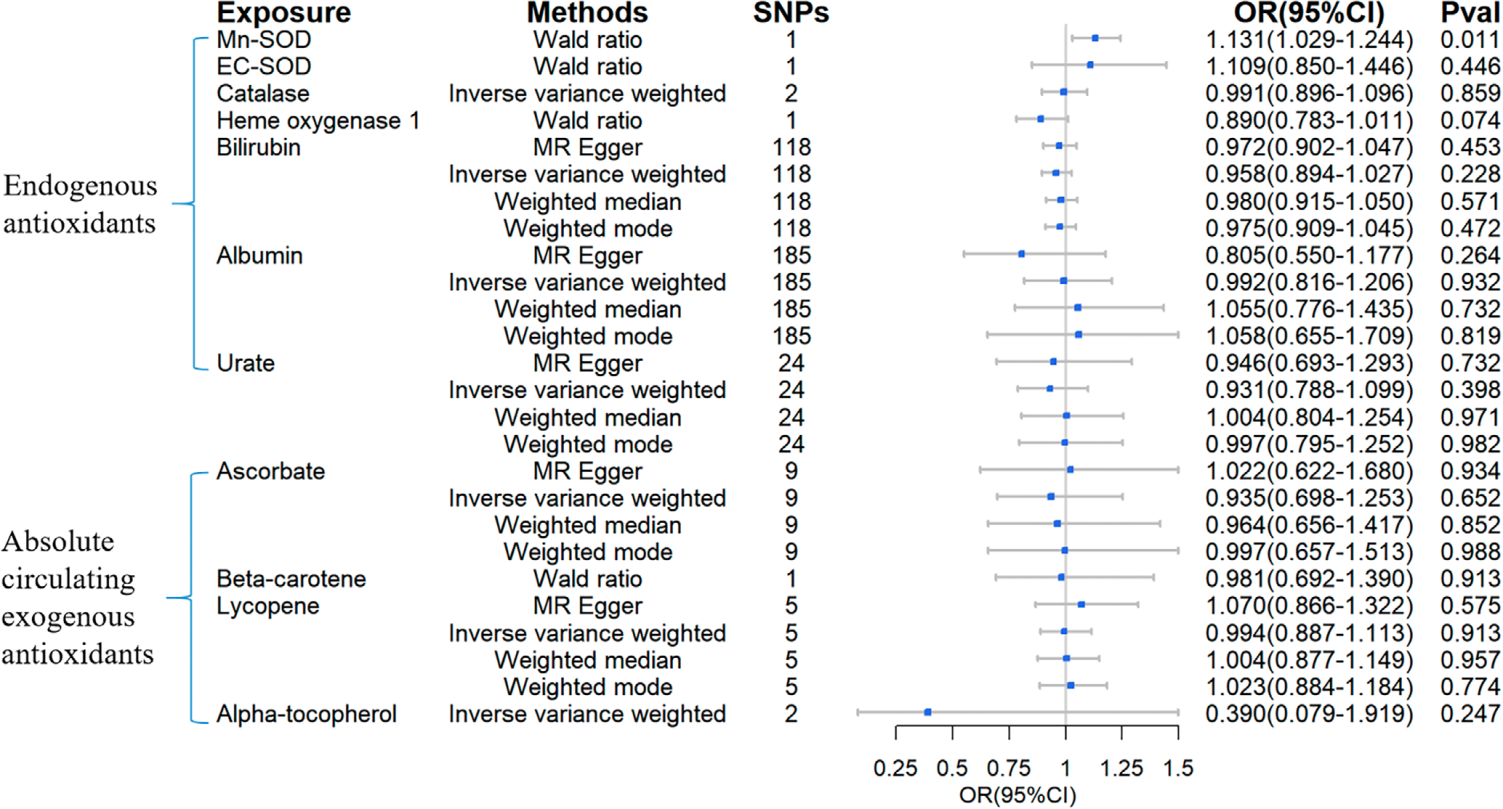
Causal estimates of forward 2SMR analyses between antioxidant levels and risks of DN. The forest plot demonstrated the 2SMR results between DN and absolute circulating antioxidants, and endogenous antioxidants, respectively. The OR for the results is depicted by the blue box of the forest plot, with the vertical lines representing the null effect (OR = 1) and the horizontal line representing the confidence interval.

Figure 2, 3, 4, 5, 6 depicted the 2SMR results for the causal associations between circulating antioxidants and the risk of T1DM, T2DM, and its complications. Among the endogenous antioxidants, higher albumin levels showed an association with a lower risk of developing T1DM (OR = 0.821, 95% CI = 0.679–0.993, Figure 2), and no significant associations were identified for Cu/Zn‐SOD, Mn‐SOD, EC‐SOD, bilirubin, catalase, HO‐1, and urate. Moreover, the 2SMR analyses indicated no significant causal links between absolute circulating levels of exogenous antioxidants and the risk of T1DM.

#### 3.1.2 Effect of Endogenous and Exogenous Antioxidant Levels on Risk of T2DM

According to the 2SMR analyses, raised bilirubin levels were linked to a reduced incidence of T2DM (OR = 0.895, 95% CI = 0.813–0.984, Figure 3), and a raised level of catalase showed a slight correlation with a lower risk of T2DM (OR = 0.965, 95% CI = 0.937–0.994, Figure 3). For exogenous antioxidants, there was a relationship between elevated β‐carotene levels and a reduced risk of T2DM (OR = 0.895, 95% CI = 0.814–0.983, Figure 3) based on the wald ratio method. No significant outcomes were observed for other antioxidants.

#### 3.1.3 Effect of Endogenous and Exogenous Antioxidant Levels on Risk of Diabetic Complications

**FIGURE 6:**
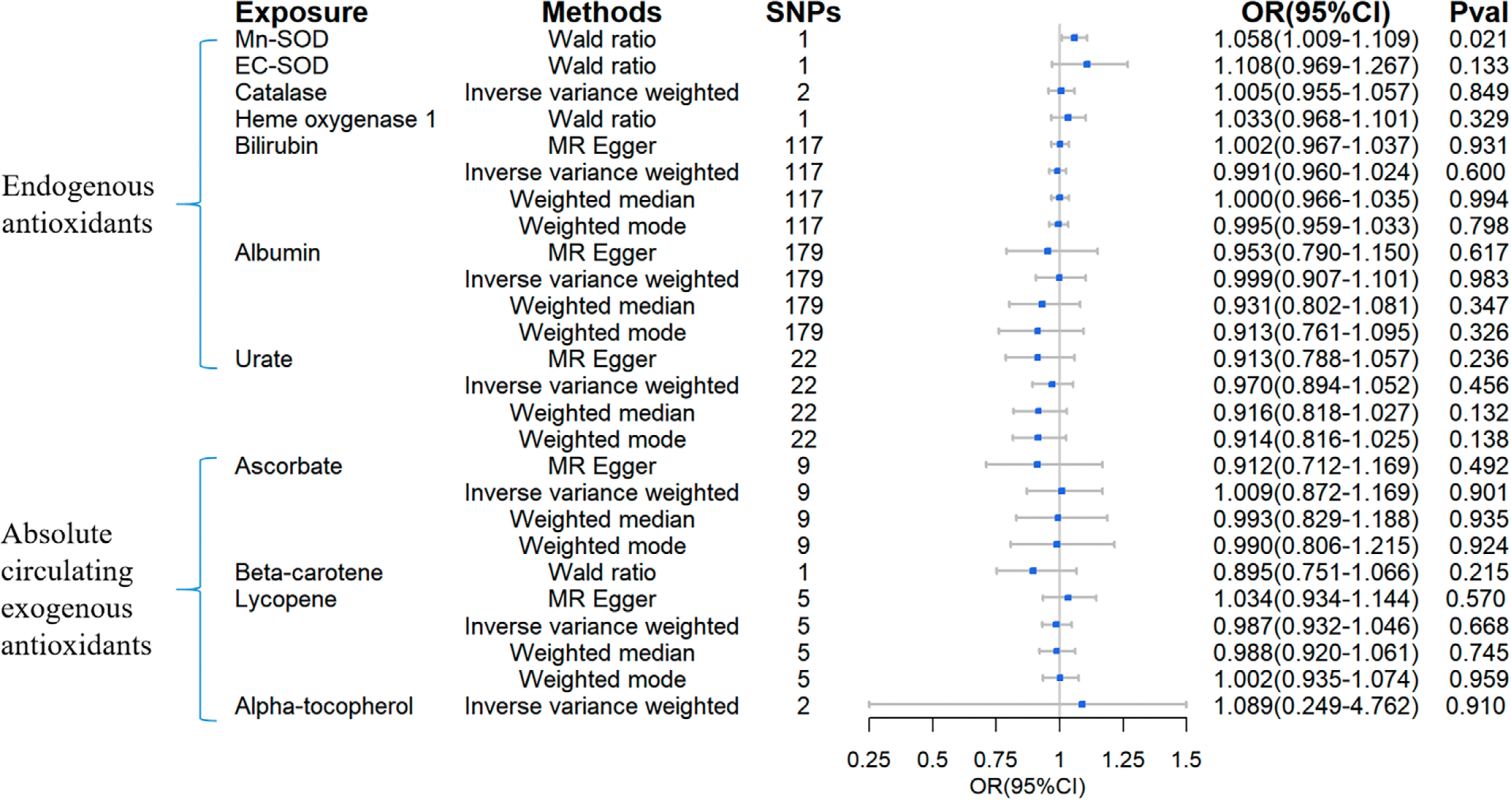
Causal estimates of forward 2SMR analyses between antioxidant levels and risks of DR. The forest plot demonstrated the 2SMR results between DR and absolute circulating antioxidants, and endogenous antioxidants, respectively. The OR for the results is depicted by the blue box of the forest plot, with the vertical lines representing the null effect (OR = 1) and the horizontal line representing the confidence interval.

The 2SMR results of the causal links between circulating antioxidant levels and the incidence of three diabetic complications were shown in the forest plot (Figures 4, 5, 6). For DK, the Wald ratio method indicated that an increased level of EC‐SOD seemed to be associated with an increased risk of this complication (OR = 1.362, 95% CI = 1.068–1.737, Figure 4). A lowered risk of DK was associated with increased levels of bilirubin (OR = 0.816, 95% CI = 0.701–0.951, Figure 4). No significant relationship was identified for other endogenous and exogenous antioxidants.

As to DN, the wald ratio method indicated that an increased level of Mn‐SOD seemed to be correlated with a heightened risk of this diabetic complication (OR = 1.131, 95% CI = 1.029–1.244, Figure 5). No significant association was observed for other endogenous and exogenous antioxidants.

As to DR, the analyses with the wald ratio method also found that Mn‐SOD seemed to be causally linked to a raised risk of this specific diabetic complication (OR = 1.058, 95% CI = 1.009–1.109, Figure 6). No significant outcomes for other included endogenous and exogenous antioxidants.

### 3.2 Outcomes of the Reverse 2SMR Analysis

**FIGURE 7:**
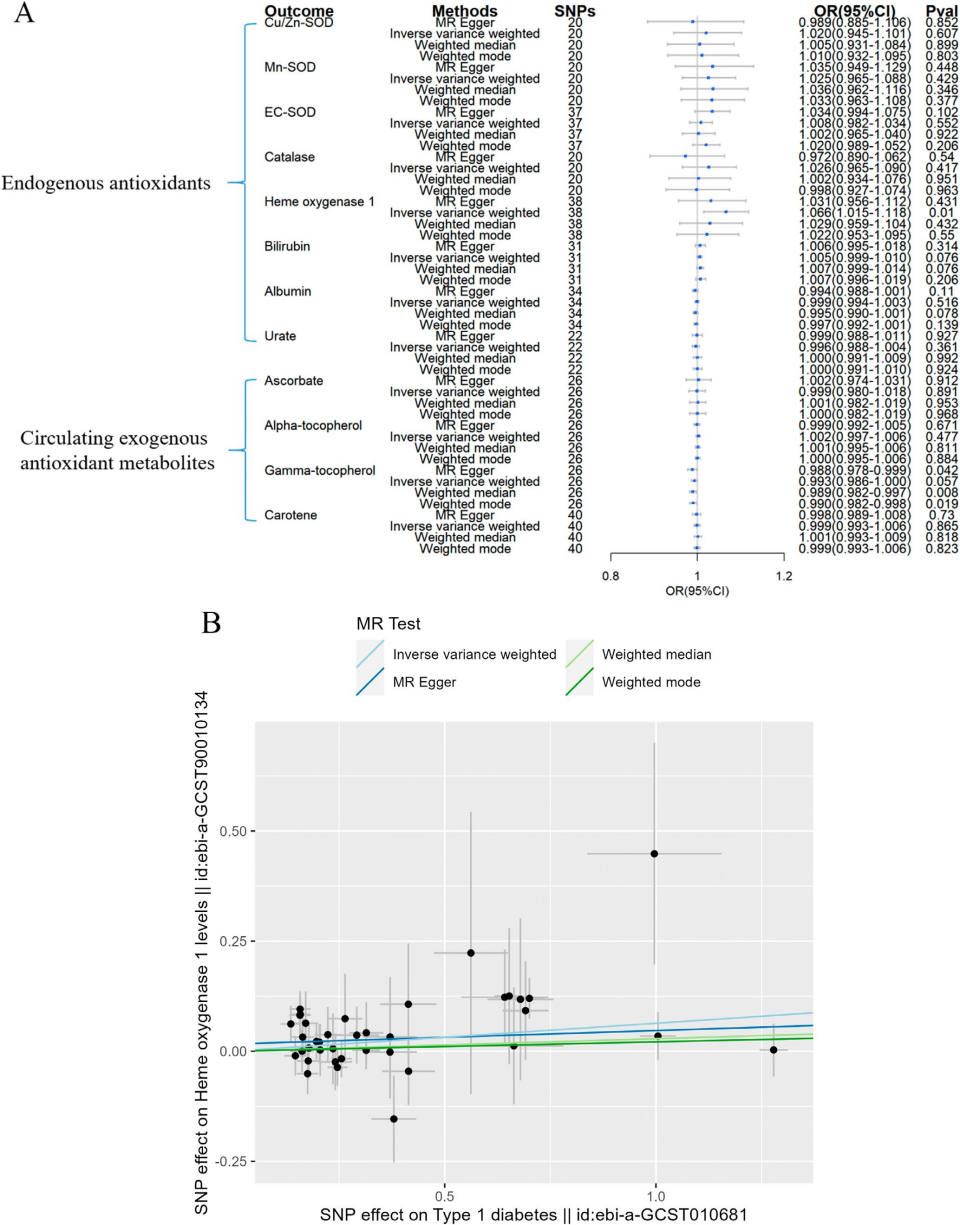
Causal estimates of reverse 2SMR analyses between antioxidant levels and risks of T1DM. (A) The forest plot demonstrated the 2SMR results between T1DM and circulating exogenous antioxidant metabolites, and endogenous antioxidants, respectively. The OR for the results is depicted by the blue box of the forest plot, with the vertical lines representing the null effect (OR = 1) and the horizontal line representing the confidence interval. (B) Scatter plot showed T1DM was causally related to elevated circulating level of HO‐1, with the slopes of the colored lines corresponding to the causal effects derived from different 2SMR analysis methods.

**FIGURE 8:**
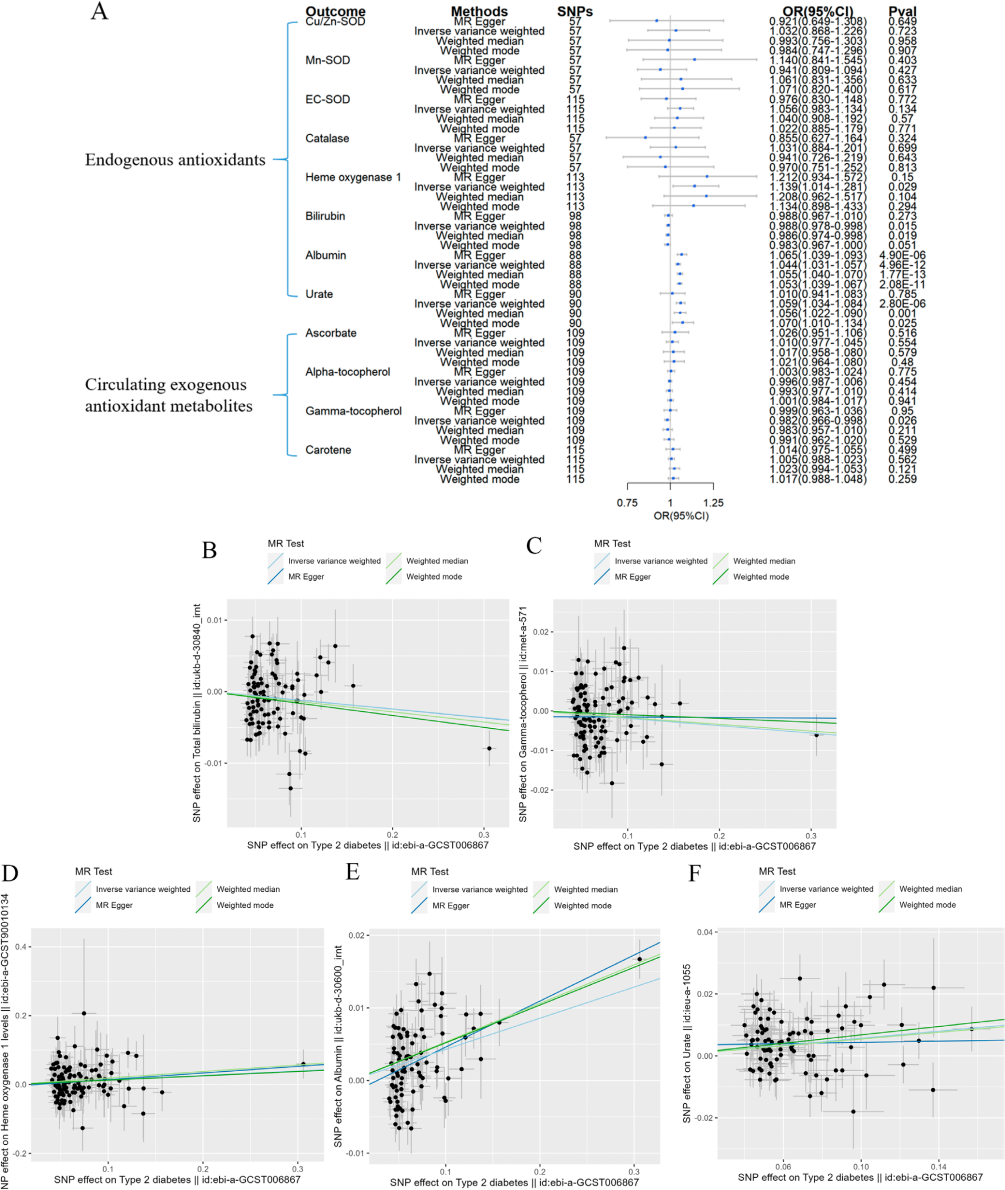
Causal estimates of reverse 2SMR analyses between antioxidant levels and risks of T2DM. (A) The forest plot demonstrated the 2SMR results between T2DM and circulating exogenous antioxidant metabolites, and endogenous antioxidants, respectively. The OR for the results is depicted by the blue box of the forest plot, with the vertical lines representing the null effect (OR = 1) and the horizontal line representing the confidence interval. The scatter plots (B–F) showed significant causal estimates in the reverse 2SMR analysis of T2DM with bilirubin, γ‐tocopherol, albumin, HO‐1 levels, and urate, respectively. The slopes of colored lines in the scatter plot correspond to the causal estimation of different 2SMR analysis methods.

Reverse MR analyses further explored the associations of T1DM, T2DM, and three diabetic complications predicting circulating levels of antioxidants. It was found that T1DM was associated with an increased circulating level of HO‐1 (OR = 1.066, 95% CI = 1.015–1.118, Figure 7). T2DM was linked to a mild reduction in bilirubin and γ‐tocopherol levels (OR = 0.988, 95% CI = 0.978–0.998 and OR = 0.982, 95% CI = 0.966–0.998, respectively, Figure 8), while slightly increased levels of HO‐1, albumin, and urate (OR = 1.139, 95% CI = 1.014–1.281, OR = 1.044, 95% CI = 1.031–1.057, and OR = 1.059, 95% CI = 1.034–1.084, respectively, Figure 8).

**FIGURE 9:**
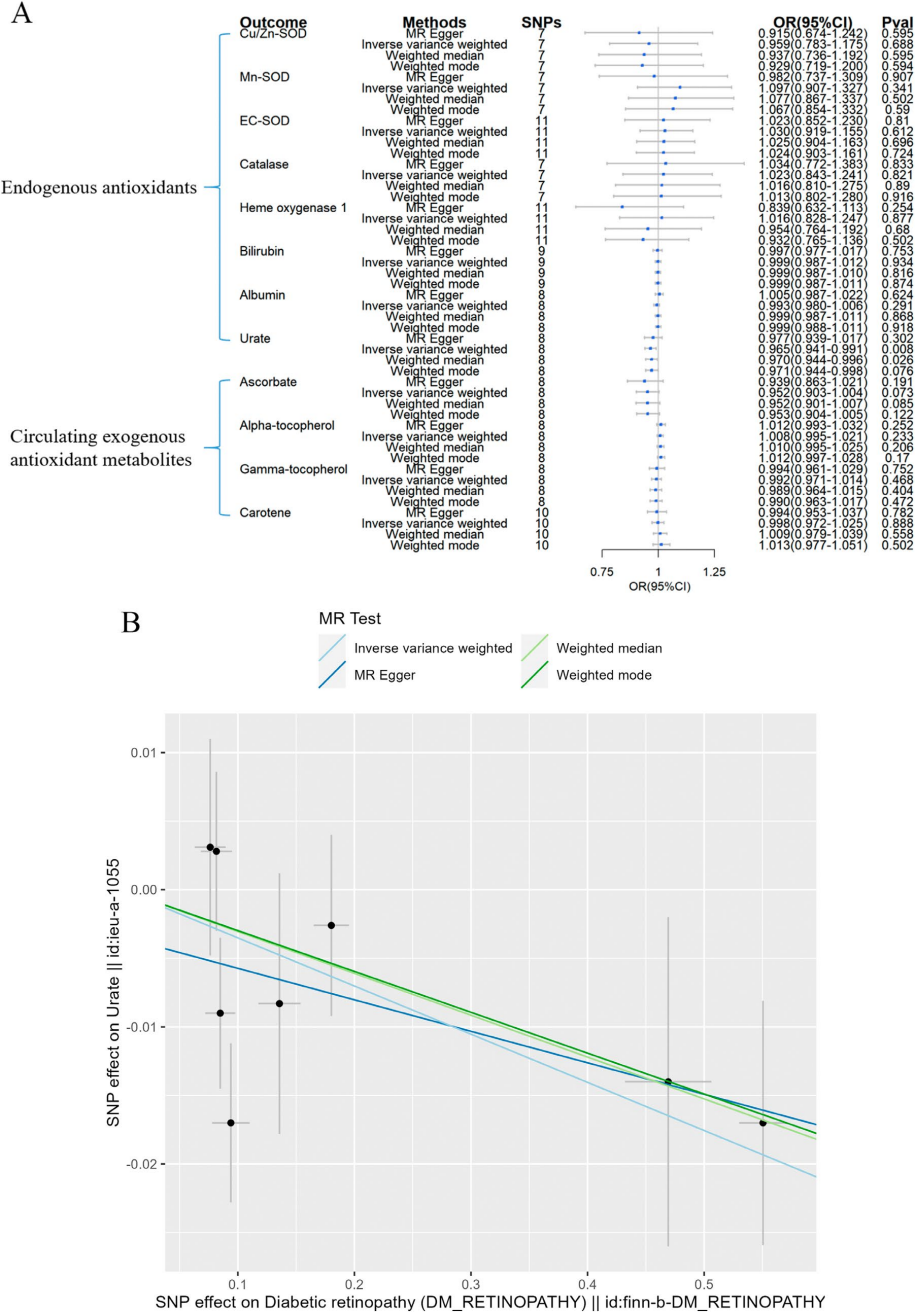
Causal estimates of reverse 2SMR analyses between antioxidant levels and risks of DR. (A) The forest plot demonstrated the 2SMR results between DR and circulating exogenous antioxidant metabolites, and endogenous antioxidants, respectively. The OR estimation of the results is represented by the blue box of the forest plot, with the vertical lines representing the null effect (OR = 1), and the horizontal line representing the confidence interval. (B) Scatter plot revealed that DR was causally linked to a slightly decreased levels of urate, with the slopes of the colored lines corresponding to the causal effects derived from different 2SMR analysis methods.

**FIGURE 10:**
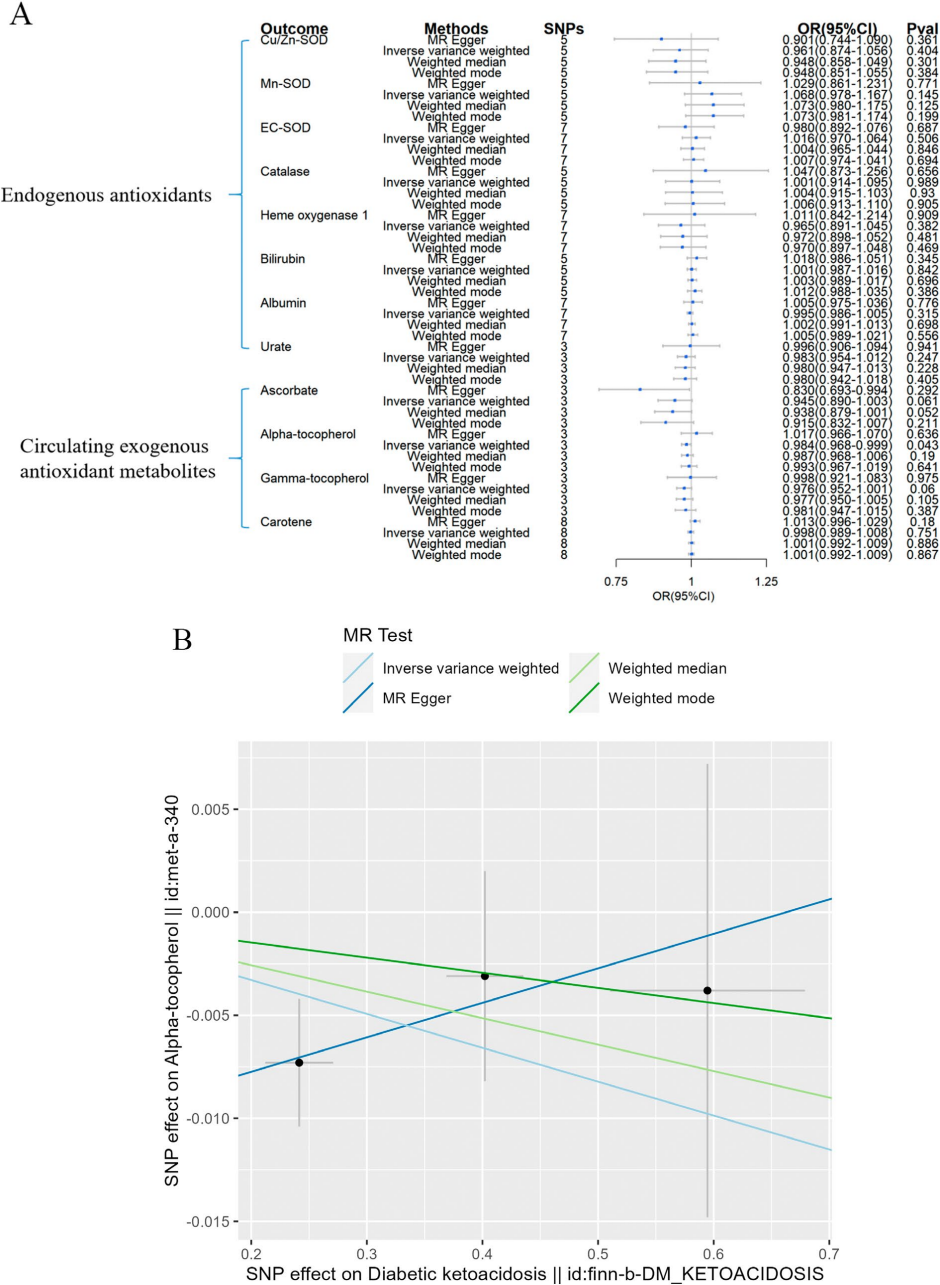
Causal estimates of reverse 2SMR analyses between antioxidant levels and risks of DK. (A) The forest plot demonstrated the 2SMR results between DK and circulating exogenous antioxidant metabolites, and endogenous antioxidants, respectively. The OR estimation of the results is represented by the blue box of the forest plot, with the vertical lines representing the null effect (OR = 1), and the horizontal line representing the confidence interval. (B) Scatter plot revealed that DK was causally linked to a slightly decreased levels of α‐tocopherol, with the slopes of the colored lines corresponding to the causal effects derived from different 2SMR analysis methods.

**FIGURE 11:**
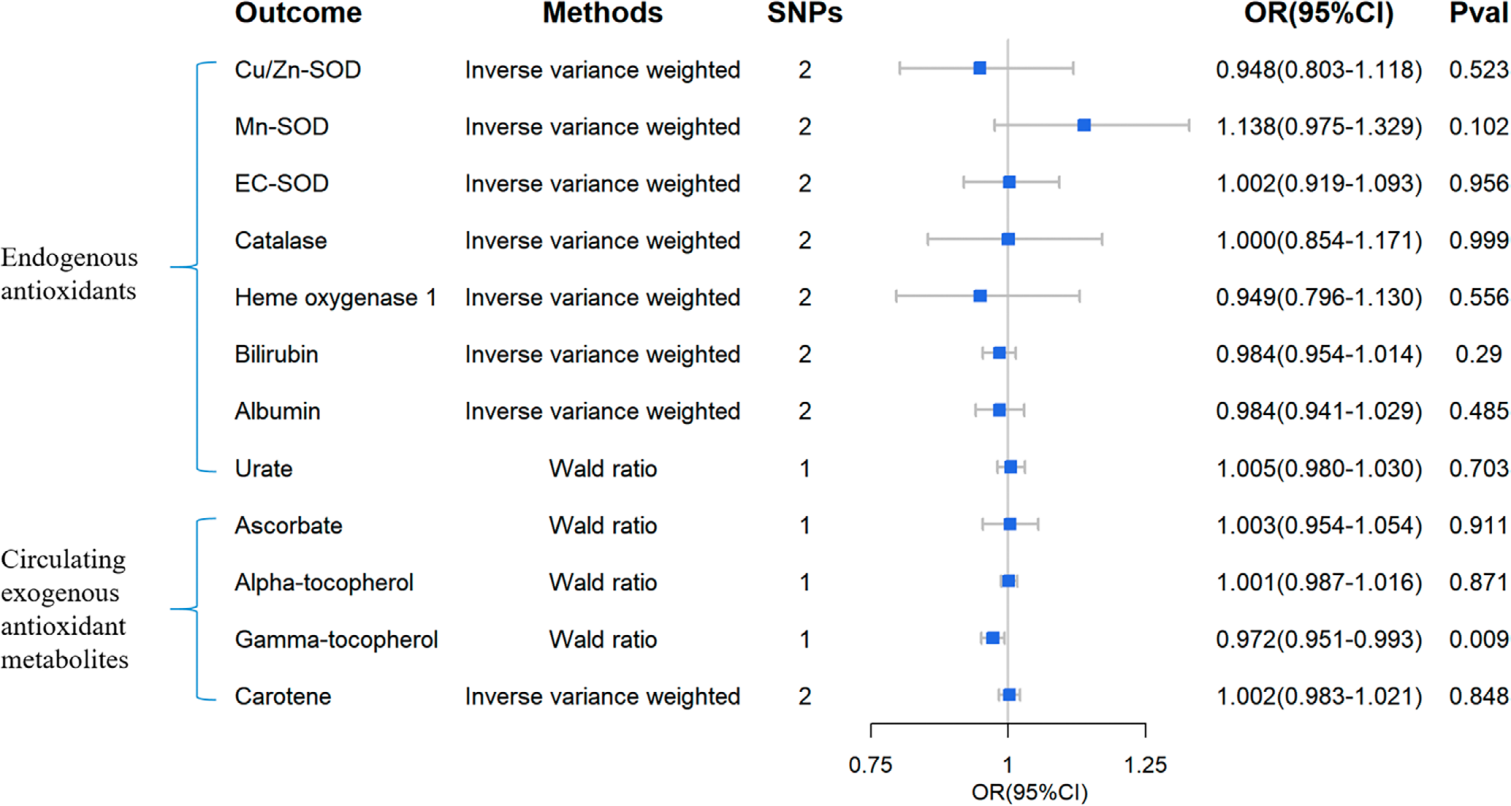
Causal estimates of reverse 2SMR analyses between antioxidant levels and risks of DN. The forest plot demonstrated the 2SMR results between DN and circulating exogenous antioxidant metabolites, and endogenous antioxidants, respectively. The OR estimation of the results is represented by the blue box of the forest plot, with the vertical lines representing the null effect (OR = 1), and the horizontal line representing the confidence interval.

The reverse 2SMR analysis outcomes of the three diabetic complications predicting antioxidant levels were shown in Figures 9, 10, 11. For endogenous antioxidants, DR showed a slight correlation with a lower levels of urate (OR = 0.965, 95% CI = 0.941–0.991, Figure 9). As to the exogenous antioxidants, DK was linked to a mild reduction in α‐tocopherol levels (OR = 0.984, 95% CI = 0.968–0.999, Figure 10), while DN was associated with slightly reduced concentrations of γ‐tocopherol (OR = 0.972, 95% CI = 0.951–0.993, Figure 11).

## 4 Discussion

**FIGURE 12:**
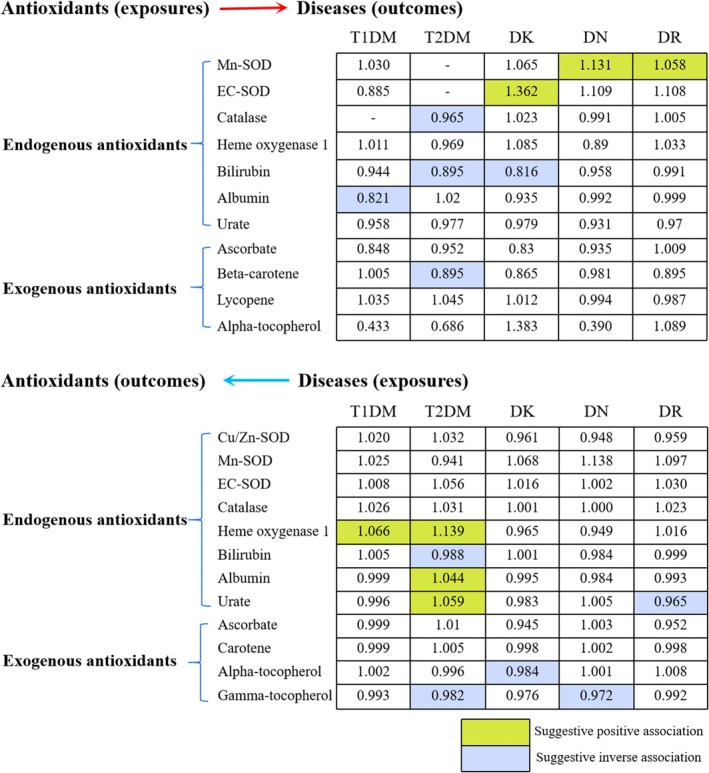
Summary of the bidirectional causal estimates between endogenous and exogenous antioxidant levels and risks of T1DM, T2DM, and its complications. The numbers within the boxes denote the ORs representing the causal associations of each exposure and outcome. Highlighted boxes indicate statistically significant causal associations between exposures and outcomes. Among them, light‐colored boxes represent suggestive inverse association, while dark‐colored boxes represent suggestive positive association.

Based on the comprehensive analyses, several suggestive antioxidants causally associated with T1DM, T2DM, and three complications were identified as shown in Figure 12. The analyses indicated that augmented levels of albumin and bilirubin exhibited a significant relationships with a lower incidence of T1DM and T2DM, respectively. Increased levels of exogenous antioxidant β‐carotene were also significantly associated with diminished risk of T2DM, which aligns with the findings observed in a recent study (Lampousi et al. 2024). For diabetic complications, it was indicated that higher bilirubin concentrations correlated with lower DK incidence. For the analysis of diabetes predicting antioxidant levels, T1DM, T2DM, and its complications were associated with varied levels of antioxidants, but the effects are weak overall.

In patients with DM, excessive production of ROS driven by hyperglycemia can induce oxidative stress in various tissues, while endogenous antioxidants, such as bilirubin, possess strong antioxidant activity and may alleviate the oxidative stress implicated in the pathology of DM (Stocker et al. 1987). Numerous studies have linked bilirubin levels to T2DM risk, while obtaining inconsistent findings about whether bilirubin is a protective antioxidant for T2DM (Lee et al. 2025; Breimer and Mikhailidis 2016; Jung et al. 2014; Benton et al. 2015). In comparison, several studies did not find the effect of bilirubin in protecting against T2DM (Wang et al. 2017). These results indicate that the observed correlation between total bilirubin levels and T2DM outcomes not only necessitates further evidence from well‐defined clinical studies but also requires MR analysis to assess the potential causal influence of bilirubin levels on T2DM risk. The present analysis supports that bilirubin may act as a protective factor against T2DM, which is consistent with previous analyses (Abbasi et al. 2015; Nano et al. 2016). Several studies have focused on exploring the association between bilirubin levels and the incidence of diabetic complications, but the outcomes are also inconsistent (Sekioka et al. 2015; Najam et al. 2014; Tafese et al. 2022; Han et al. 2010; Abe et al. 2021; Al‐Suhaimi and Al‐Rubaish 2024). Our analyses support that an elevated level of bilirubin shows a correlation with a reduced risk of DK.

Additionally, as a significant component of the antioxidative system, numerous studies have examined the impacts of exogenous antioxidant supplementation on risks of DM. For instance, a strong association between increased serum β‐carotene concentrations and decreased risk of T2DM was detected in a longitudinal community‐based study (Arnlöv et al. 2009). Furthermore, a comprehensive meta‐analysis study suggested an association between total carotenoids with increased intake and elevated circulating concentrations, particularly β‐carotene, and reduced incidence of T2DM, while whether the association is causal remains to be studied (Jiang et al. 2021). The present analysis supports that an increased level of β‐carotene was significantly associated with a decreased risk of T2DM. Further research is required to reach definitive conclusions concerning the impact of other exogenous antioxidant supplementation on DM and its complications.

We also performed the reverse analysis of diabetes and its complications predicting antioxidant levels. As far as we are aware, this is the first‐ever MR analysis on diabetes predicting antioxidant levels. Despite DM and its complications being likely associated with varied levels of several antioxidants, the associations seem to be weak overall.

## 5 Strengths and Limitations

We would like to draw attention to the strengths and weaknesses of the current study. Several strengths of our research are worth noting. First, we analyzed causality bidirectionally between antioxidants and risks of T1DM, T2DM, and diabetic complications. Second, *both* a series of *endogenous and* exogenous *antioxidant levels are considered to assess their association with the risk of* T1DM, T2DM, and three complications.

Certainly, some limitations of the current study should also be noted. First of all, the present study limited the European population of the included studies, and thus cannot ensure other differences that might affect the risk of DM and its complications. Moreover, subgroup and hierarchical analysis cannot be carried out according to age, sex, and other characteristics. The number of valid genetic instruments for partial exposure factors was limited by available GWAS data, potentially reducing the statistical power of our analyses. Although the exposure and outcome datasets involved in this paper were derived from independent cohorts, potential cryptic sample overlapmay lead to deviation in the degree of relationship between exposure and outcome. In addition, some causal estimates in this study exhibit high heterogeneity in sensitivity analysis, which may arise from small effect sizes of the exposure on the outcome, insufficient sample size of the outcome GWAS, or a low number of genetic instruments. Consequently, conclusions should be drawn with greater caution.

## 6 Conclusion

In summary, the analyses indicated the likely protective effects of *endogenous* bilirubin and albumin against T1DM, T2DM, and DK, respectively. Increased absolute level of exogenous antioxidant β‐carotene is also likely to be correlated with decreased risk of T2DM. Additional studies are required to validate the findings and to explore the potential applications for the prevention of DM and its complications, or their prediction.

## Author Contributions

L.S. and H.‐F.J. conceived and designed the project. L.S. and L.M. collected the data. L.S. and L.M. performed the calculations. L.S. and H.‐F.J. interpreted the results. L.S. and H.‐F.J. drafted and revised the manuscript. All authors read and agreed on the final version of the manuscript.

## Conflicts of Interest

The authors declare no conflicts of interest.

## Data Availability

The data that support the findings of this study are available on request from the corresponding author.
